# Electrochemically Deposited Zinc (Tetraamino)phthalocyanine as a Light-activated Antimicrobial Coating Effective against *S. aureus*

**DOI:** 10.3390/ma15030975

**Published:** 2022-01-27

**Authors:** Ivan Gusev, Marli Ferreira, Davy-Louis Versace, Samir Abbad-Andaloussi, Sandra Pluczyk-Małek, Karol Erfurt, Alicja Duda, Przemysław Data, Agata Blacha-Grzechnik

**Affiliations:** 1 Faculty of Chemistry, Silesian University of Technology, Strzody 9, 44-100 Gliwice, Poland; ivan.gusev@polsl.pl (I.G.); sandra.pluczyk-malek@polsl.pl (S.P.-M.); karol.erfurt@polsl.pl (K.E.); alicjaa.duda@gmail.com (A.D.); 2 Centre for Organic and Nanohybrid Electronics, Silesian University of Technology, Konarskiego 22b, 44-100 Gliwice, Poland; marli.ferreira@polsl.pl (M.F.); przemyslaw.data@polsl.pl (P.D.); 3 Institut de Chimie et des Matériaux Paris-Est (ICMPE, UMR-CNRS 7182-UPEC), 2-8 Rue Henri Dunant, 94320 Thiais, France; 4 Laboratoire Eau, Environnement, Systèmes Urbains (LEESU), UMR-MA 102, Université Paris-Est Créteil (UPEC), 61 Avenue Général de Gaulle, 94010 Créteil Cedex, France; abbad@u-pec.fr

**Keywords:** electrochemical deposition, light-activated antimicrobial layer, reactive oxygen species (ROS), phthalocyanines, photosensitizers

## Abstract

Light-activated antimicrobial coatings are currently considered to be a promising approach for the prevention of nosocomial infections. In this work, we present a straightforward strategy for the deposition of a photoactive biocidal organic layer of zinc (tetraamino)phthalocyanine (ZnPcNH_2_) in an electrochemical oxidative process. The chemical structure and morphology of the resulting layer are widely characterized by microscopic and spectroscopic techniques, while its ability to photogenerate reactive oxygen species (ROS) is investigated in situ by UV–Vis spectroscopy with α-terpinene or 1,3-diphenylisobenzofuran as a chemical trap. It is shown that the ZnPcNH_2_ photosensitizer retained its photoactivity after immobilization, and that the reported light-activated coating exhibits promising antimicrobial properties towards *Staphyloccocus aureus* (*S. aureus*).

## 1. Introduction

In recent years, thin layers of organic and/or inorganic photosensitizers have gained wide research interest for application as antimicrobial coatings deposited on the surfaces of objects that are used daily. The main advantage of such structures is their remarkable biocidal efficiency against bacteria, viruses, and fungi. This makes them an attractive alternative to classical antimicrobial coatings containing poly(ethylene glycol) chains, silver or copper nanoparticles, quaternary ammonium salts or cations, fluorinated polymers, etc. [1,2]. The antimicrobial action of photoactive layers is based on the formation of so-called reactive oxygen species (ROS), including superoxides, peroxides, and singlet oxygen [3]. ROS act in a highly effective and non-selective manner (e.g. via the oxidation of enzymes, by increasing ions’ permeability by the cell wall) against microorganisms, which, in turn, strongly reduces the possibility of the development of resistance by microbes [4,5,6]. Until now, light-activated antimicrobial coatings containing, e.g., phenothiazine, porphyrin, or fullerene photosensitizers have been successfully applied against *Staphylococcus aureus, Escherichia coli, Clostridium difficile, Candida albicans*, and *Pseudomonas aeruginosa* [7,8,9,10,11,12,13,14]. However, the practical usage of such photoactive layers is quite limited, and intense research is conducted in order to overcome key constraints, such as (photo)stability, long-term action, or the ease and accessibility of a deposition procedure.

For many years, phthalocyanines (Pcs) have been mainly investigated as promising candidates for application in (opto)electronics. Until recently, Pcs in a free-base form or with a central metal have been used in organic photovoltaic devices (OPVs) [15,16,17], organic light-emitting diodes (OLEDs) [18,19], or gas sensors [20,21,22]. Their effectiveness as photoinitiating systems [23,24] and photosensitizers [25,26] has also been reported. Moreover, it has been shown that the photosensitizing abilities of Pcs can be tuned by changing the central metal atom and/or by the introduction of outer functional groups.

Taking into account the above, in this work, we aimed to investigate the possibility of applying an electrodeposited Pc-based photoactive layer as a light-activated antimicrobial coating. Thus, zinc (tetraamino)phthalocyanine (ZnPcNH_2_) was selected as a primary photosensitizer molecule. The choice was first made based on the fact that Zn-containing Pcs show considerable efficiency of ROS production [27,28] and high antimicrobial properties [12,29]. Secondly, as shown in our previous work, the outer primary amino groups can be used in the electrochemically driven deposition of Pcs [30]. The presented novel, straightforward strategy, consisting of an electrochemical oxidation of primary amino groups present in the ZnPcNH_2_ structure, may be beneficial for the formation of a layer on conductive substrates. The deposited coating is widely characterized by means of spectroscopic and microscopic techniques. It is shown that the immobilized ZnPcNH_2_ photosensitizer is able to produce ROS under red laser or white light illumination and thus shows a light antimicrobial response against *Staphyloccocus aureus* (*S. aureus*).

## 2. Materials and Methods

### 2.1. Materials

Zinc (tetraamino)phthalocyanine (ZnPcNH_2_) was synthesized based on previous works [31,32]. The synthesis procedure and the identification of the obtained product are described in the Supporting Information.

Dimethylformamide (DMF; ≥99.8%, Sigma Aldrich, St. Louis, MO, USA) containing tetrabutylammonium tetrafluoroborate (TBABF_4_; 99%, Sigma Aldrich, St. Louis, MO, USA) was used as an electrolyte solution for the electrochemical deposition of (ZnPcNH_2_)_layer_. Indium tin oxide/borosilicate glass (ITO) purchased from Präzisions Glas & Optik GmbH (PGO, Iserlohn, Germany) was used as a support. ROS photogeneration was investigated in α-terpinene (TCI; purity > 90%)–acetonitrile (Sigma Aldrich, St. Louis, MO, USA) or 1,3-diphenylisobenzofuran (DPBF; Acros Organics, Geel, Belgium, purity > 97%)–methanol (Acros Organics, Geel, Belgium, 99.9%) systems.

### 2.2. Formation and Characterization of (ZnPcNH_2_)_layer_

(ZnPcNH_2_)_layer_ was formed on ITO or a platinum plate from 0.1 mM solution of ZnPcNH_2_ in 0.1 M TBABF_4_/DMF. The starting solution was homogenized with an ultrasonic mixer and purged with argon for 15 minutes to remove oxygen. The process of electrochemical deposition was conducted using a cyclic voltammetry (CV) technique and a CHI 660C electrochemical workstation (CH Instruments Inc., Austin, TX, USA). A three-electrode setup was used: ITO or Pt as a working electrode, Ag wire as a pseudo-reference electrode, and glassy carbon (GC) as a counter electrode. The electrodes were copiously rinsed with DMF and mounted in a Teflon holder prior to use. The electrochemical deposition was conducted by means of cyclic voltammetry (CV) with the following process parameters: the potential range (−1.8; 1.4) V, 10 scan cycles, and a scan rate of 0.1 V/s. Ferrocene (Fc/Fc^+^) was used as a reference for the potential calibration.

The morphology of the layer was investigated using scanning electron microscopy (SEM) and atomic force microscopy (AFM). SEM images were acquired using Phenom ProX with an accelerating voltage equal to 15 kV and a magnification between 15,000× and 20,000×. The samples were sputter coated with 10 nm of gold film (Q150R Quorum Technologies, Laughton, East Sussex, UK) before SEM imaging. The images were analyzed with Phenom software. AFM investigations were conducted using Nanosurf CoreAFM working in a contact mode with the standard contact-mode AFM HQ:CSC17/Al BS (MikroMasch, Tallinn, Estonia) probe (resonance frequency 13 kHz; force constant 0.18 Nm^−1^). The images were processed with the use of Gwyddion SPM (Brno University of Technology, Brno, Czech Republic).

UV–Vis spectra of the ZnPcNH_2_ solution in DMF and (ZnPcNH_2_)_layer_ supported on ITO were acquired with a Hewlett Packard 8452A UV–Vis spectrometer. IR spectra of ZnPcNH_2_ powder and (ZnPcNH_2_)_layer_ deposited on a platinum plate were recorded within the 3500–700 cm^−1^ range in attenuated total reflectance (ATR) mode using a Perkin Elmer Spectrum Two IR spectrometer (Hopkinton, MA, USA).

### 2.3. Reactive Oxygen Species (ROS) Photogeneration and Microbiological Analysis

The process of ROS photogeneration was investigated either under 638 nm diode laser (Oxxius LBX-638-150-ELL-PP, Lannion, France, power reduced to 20 mW) or white light (Fiber-Coupled Xenon Light Source, Newton, NJ, USA, SLS205, 75 W, Thorlabs) illumination. In the first case, a DPBF trap was used with 0.05 mM solution in methanol, while in the second case, the trap was used with 0.05 mM solution of α-terpinene in acetonitrile. The experimental setup was arranged in this way so that a photoactive layer supported on a glass plate was put in a quartz cuvette (Hellma Analytics, Müllheim, Germany, 10 × 4 mm). It was illuminated with a light source, while UV–Vis spectra of the chemical traps were collected in situ with a Hewlett Packard 8452A UV–Vis spectrometer (Palo Alto, CA, USA) along a direction perpendicular to the layer’s illumination. The course of ROS production was observed by a drop of DPBF or α-terpinene absorbance at 410 nm or 266 nm, respectively.

The antibacterial properties of the ZnPcNH_2_-derived coatings, glass, and ITO surfaces were evaluated against *S. aureus* ATCC12000 according to previously described procedures [13,33,34]. The corresponding coatings were immersed in a bacterial solution for 24 h prior to visible-light activation, in order to maximize bacterial adhesion on surfaces. Then, half of the samples were kept in the dark while half of them were illuminated for 1 h on each side under a solar emission lamp. Following adhesion, all the defined square samples were rinsed seven times with sterile NaCl solution (0.9% *w*/*v*) to remove the non-adherent or dead bacteria from each surface. Then, samples were immersed in 3 mL of sterile saline solution and sonicated for 5 min in order to detach the viable bacteria from the surface of the studied samples. This solution was serially diluted by 10 to 10^5^ factors. An amount of 100 µL of each diluted and detached microorganism solution was then introduced on the surface of a Plat Count agar plate. This process was repeated as many times as there were dilutions. The total amount of viable bacteria was determined by counting the colony-forming units, after 48 h of incubation of the agar plates at 37 ° C (for each dilution), and levels of adhesion were given as numbers of counted bacteria/cm^2^ (cm^2^ corresponds to the surface of the defined samples). Four experiments were conducted on each sample.

## 3. Results and Discussion

### 3.1. Formation and Spectroscopic and Microscopic Characterization of (ZnPcNH_2_)_layer_

**Figure 1:**
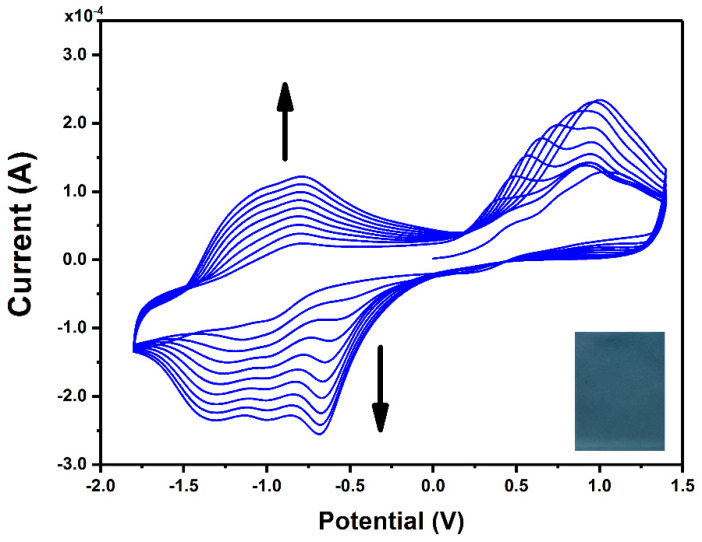
CV curves recorded for the ITO working electrode in 0.1 mM ZnPcNH_2_ electrolyte solution (0.1 M TBABF_4_/DMF). Inset: photography of (ZnPcNH_2_)_layer_ electrodeposited on ITO.

Figure 1 presents CV curves recorded during continuous scanning in an electrolyte solution containing ZnPcNH_2_. In the first anodic scan, an irreversible oxidation can be observed at ca. 0.75 V vs. Ag, which can be assigned to the oxidation of the primary amino group of ZnPcNH_2_ to a radical cation, similar to the electro-oxidation of the aniline monomer [35]. A steady increase in the registered current in the broad potential range during continuous scanning can be observed, confirming the formation of the electroactive layer on the surface of the ITO electrode. The newly occurring redox couples at ca. −0.75 V and −1.25 V arise from the electrochemical activity of the ZnPc layer [36,37,38,39]. In the course of the 10 scan cycles of the electrodeposition process, a uniform blue-green (ZnPcNH_2_)_layer_ was synthesized on the ITO surface (Figure 1 inset).

**Figure 2:**
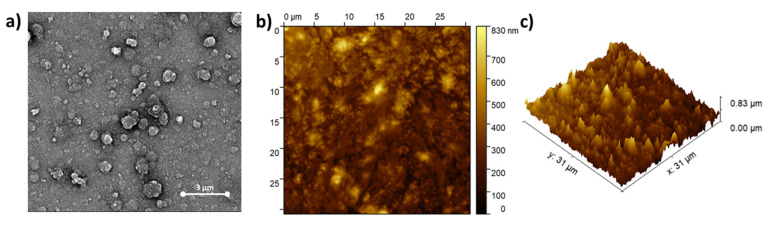
(**a**) SEM and (**b**,**c**) AFM images of (ZnPcNH_2_)_layer_ electrodeposited on ITO.

The morphology of (ZnPcNH_2_)_layer_ on ITO was investigated using SEM and AFM techniques. An SEM image of ITO covered with ZnPcNH_2_ is shown in Figure 2a. Spherical crystallites, typical for solution-deposited ZnPc films [40,41], sized within the 0.6–1.3 µm range, can be observed. Further, AFM studies confirmed the continuous coverage of the ITO surface, as shown in the exemplary AFM topography 31 × 31 µm^2^ image shown in Figure 2b. An AFM image shown in Figure 2c also confirms the presence of rather vertically oriented crystallites. This is in contrast to ZnPc films deposited on a heated substrate with the PVD technique which have a rather ribbon-like structure [42]. The root mean square roughness (RMS) value for the presented area of the film was 100.9 nm.

**Figure 3:**
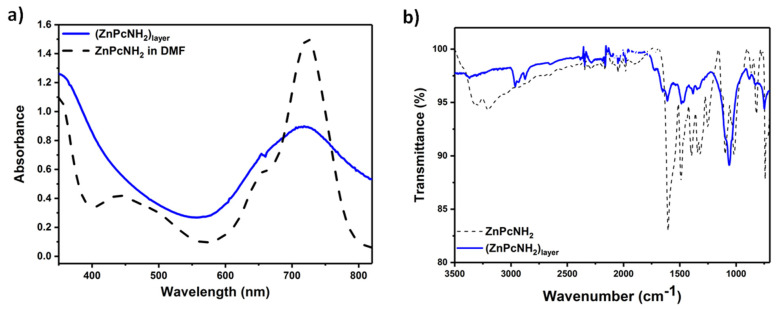
(**a**) UV–Vis of ZnPcNH_2_ (black dashed line) and (ZnPcNH_2_)_layer_ (blue line); (**b**) ATR-IR spectra of ZnPcNH_2_ (black dashed line) and (ZnPcNH_2_)_layer_ (blue line).

The optical properties and the chemical structure of (ZnPcNH_2_)_layer_ were characterized by UV–Vis and IR spectroscopies, respectively. UV–Vis spectra of the solution of ZnPcNH_2_ in DMF and (ZnPcNH_2_)_layer_ deposited on ITO are presented in Figure 3a. The characteristic B (Soret band) and Q bands (π *→* π* transition band) for ZnPcNH_2_, both in a solution phase and in the form of a layer, can be observed at ca. 350 nm and 720 nm, respectively. The latter is significantly broadened in the case of the layer, possibly due to the aggregation of ZnPc molecules [36,43,44]. Notably, the deposited Pc layer exhibits strong and broad absorption in the visible range, which should be advantageous for a white light-driven photosensitization process.

Figure 3b presents the ATR-IR spectra recorded for the ZnPcNH_2_ powder and (ZnPcNH_2_)_layer_ deposited on a Pt plate. Both spectra reveal the presence of the characteristic stretching vibrations of H-C bonds within Pc’s ring at ca. 2950 cm^−1^ [45]. Next, the C-N and C-C stretching vibrations in the isoindole part can be observed at 1260 ± 5 and 1327 ± 10 cm^−1^, respectively [45], while stretching vibrations of the Zn-N bond can be observed at 820 ± 6 cm^−1^ [46], and out-of-plane bending of C-H bonds at 735 cm^−1^ [47]. The presence of the metal-free Pc in the structure of the layer can be excluded based on the absence of N-H vibrations within the Pc ring that typically occur at ca. 1000 cm^−1^ [47]. Finally, the clear difference between spectra of the monomer and the layer can be seen above 3000 cm^−1^, where the stretching vibrations of amino groups occur [48]. In the case of ZnPcNH_2_, two bands at 3203 and 3304 cm^−1^ are present that are significantly less intense in the case of (ZnPcNH_2_)_layer_. This confirms that the primary amino groups are involved in the process of the layer’s electrodeposition. The full assignment of the observed peaks is presented in Table S1.

### 3.2. Reactive Oxygen Species (ROS) Photogeneration and Antimicrobial Properties of (ZnPcNH_2_)_layer_

**Figure 4:**
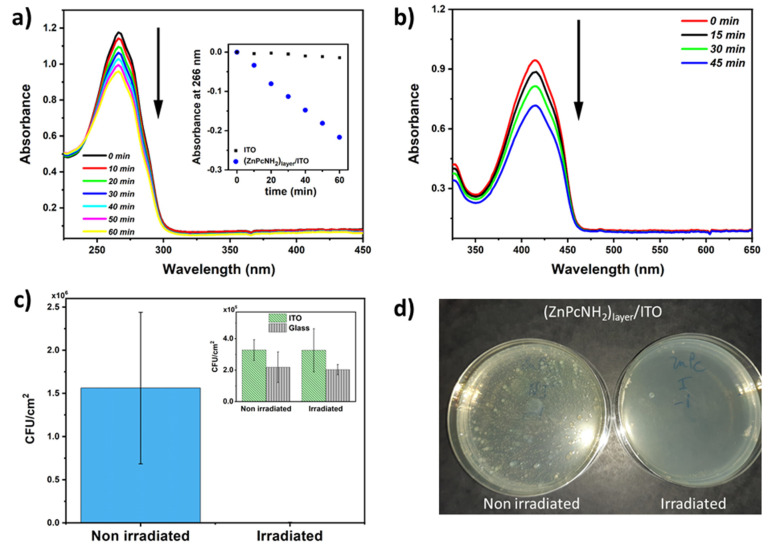
(**a**) UV–Vis spectra of α-terpinene (ACN solution) recorded during illumination of (ZnPcNH_2_)_layer_; inset: a drop in absorbance at 266 nm during illumination of unmodified ITO and (ZnPcNH_2_)_layer_/ITO. (**b**) UV–Vis spectra of DPBF (methanol solution) recorded during illumination of (ZnPcNH_2_)_layer_. (**c**) Evolution of colony-forming units, CFU/cm^2^ (for *S. aureus*), at the surface of the non-irradiated and irradiated (ZnPcNH_2_)_layer_/ITO; inset: ITO and glass, *p* < 0.001, *n* = 4. (**d**) Optical images of *S. aureus* colonies on Petri dish after 48 h of incubation at 37 °C that adhered to the (ZnPcNH_2_)_layer_/ITO surface with and without irradiation.

In the next step, the process of ROS photogeneration by the electrodeposited Pc layer was investigated under white light illumination, using indirect ROS detection employing UV–Vis spectroscopy to follow the changes in the concentration of α-terpinene, i.e., a chemical trap [49]. An exemplary UV–Vis spectra of α-terpinene in ACN recorded in the course of the illumination of (ZnPcNH_2_)_layer_/ITO are shown in Figure 4a. A successive decrease in the absorbance of α-terpinene at 266 nm can be observed, when (ZnPcNH_2_)_layer_ is illuminated. Notably, the drop is significantly higher than in the case of uncovered ITO (Figure 4a, inset). This confirms that the chemical trap reacts with ROS produced by the Pcs-containing layer [50]. Importantly, since no new band arises in the UV–Vis spectra during the photoprocess, it can be stated that (ZnPcNH_2_)_layer_ is stable and that the immobilized photosensitizers are not released into the environment.

Additionally, the photogeneration of ROS by (ZnPcNH_2_)_layer_ was confirmed with a DPBF trap. In this case, a 638 nm diode laser was used as an illumination source because DPBF is not stable under white light [51]. As shown in Figure 4b, a clear drop in the absorbance at 410 nm can be observed, which is associated with a reaction of DPBF with the generated ROS. Similar to the acetonitrile solution, the photoactive layer remains stable in contact with methanol.

The capability of the ZnPcNH_2_-derived coating to inhibit *S. aureus* adhesion was assessed with and without visible-light illumination and compared with reference samples, i.e., ITO and glass surfaces (Figure 4c). Prior to anti-adhesion experiments, samples were incubated in a *S. aureus* solution for 24 h to allow optimal adhesion of *S. aureus* on coatings. Interestingly, when the incubated coatings were illuminated under visible light, a tremendous inhibition of the bacterial adhesion was observed on the ZnPc-containing coating: *S. aureus* inhibition under light reached up to 99.8% (Figure 4d). Importantly, visible light has no influence on the adhesion or proliferation of *S. aureus* on ITO and glass surfaces (Figure 4c, inset). These interesting results can be easily explained by the production of reactive oxygen species at the surface of the phthalocyanine-derived coatings under light activation, as previously described. These results point out the high potentiality of photoactivated coatings for disinfection applications.

## 4. Conclusions

In this work, ZnPcNH_2_ photosensitizer was deposited on ITO/glass surface in a straightforward electrochemical process that involved an oxidation of the outer primary amino groups present in the Pc structure. The chemical structure of the formed layer was confirmed by ATR-IR spectroscopy. It was shown that (ZnPcNH_2_)_layer_ possesses several advantages such as broadband absorption in the visible region and high environmental stability, even in organic solvents. The resulting coating can be activated by white light illumination to effectively produce reactive oxygen species. This proves that immobilized ZnPcNH_2_ retains its photosensitizing properties. Finally, it was shown that (ZnPcNH_2_)_layer_ is active against *S. aureus*, there was a 99.8% light-associated decrease in the number of the adhered bacteria. To sum up, the presented results confirm that with a proper design of a photosensitizer molecule’s structure, an efficient light-activated antimicrobial layer can be deposited in a simple electrodeposition process. In this work, ITO/glass or platinum was used as a support. However, it is believed that, since the deposition process is conducted in an oxygen-free organic environment under a rather low oxidative potential, it may be attractive for covering various (semi)conductive materials, irrespective of size or shape.

## Data Availability

Data are presented in the article and Supplementary Materials.

## Supplementary Materials

The following are available online at https://www.mdpi.com/article/10.3390/ma15030975/s1: Synthesis and characterization of ZnPcNH_2_ monomer, Table S1: Assignment of IR signals for ZnPcNH_2_ monomer and (ZnPcNH_2_)_layer_.
