# Supplementary material for: Electrochemically Deposited Zinc (Tetraamino)phthalocyanine as a Light-activated Antimicrobial Coating Effective against S. aureus
Source: Materials (Basel). 2022 Jan 27;15(3):975. doi: 10.3390/ma15030975 (PMC8838431; doi:10.3390/ma15030975)
Supplement: Supplementary file 1 [file materials-15-00975-s001.zip › materials-1531679-supplementary.pdf]

Supporting information

Electrochemically deposited zinc (tetraamino)phthalocyanine as a light-activated antimicrobial coating effective against *S. aureus*

Ivan Gusev [1,2], Marli Ferreira [2], Davy-Louis Versace [3*], Samir Abbad-Andaloussi [4], Sandra Pluczyk-Małek [1,2], Karol Erfurt [1], Alicja Duda [1], Przemyslaw Data [2] and Agata Blacha-Grzechnik [1,2*]

1 Faculty of Chemistry, Silesian University of Technology, Strzody 9, 44-100 Gliwice, Poland; ivan.gusev@polsl.pl, marli.ferreira@polsl.pl, sandra.pluczyk-malek@polsl.pl, karol.erfurt@polsl.pl, alicjaa.duda@gmail.com, przemyslaw.data@polsl.pl, agata.blacha@polsl.pl

2 Centre for Organic and Nanohybrid Electronics, Silesian University of Technology, Strzody 9, 44-100 Gliwice, Poland

3 Institut de Chimie et des Matériaux Paris-Est (ICMPE, UMR-CNRS 7182-UPEC), 2-8 rue Henri Dunant, 94320 Thiais, France; davy-louis.versace@u-pec.fr

4 Université Paris-Est Créteil (UPEC), Laboratoire Eau, Environnement, Systèmes Urbains (LEESU), UMR-MA 102, 61 avenue Général de Gaulle, 94010 Créteil Cedex, France; abbad@u-pec.fr

* Correspondence: agata.blacha@polsl.pl, davy-louis.versace@u-pec.fr

1. Synthesis of zinc (tetraamino)phthalocyanine (ZnPcNH$_2$)

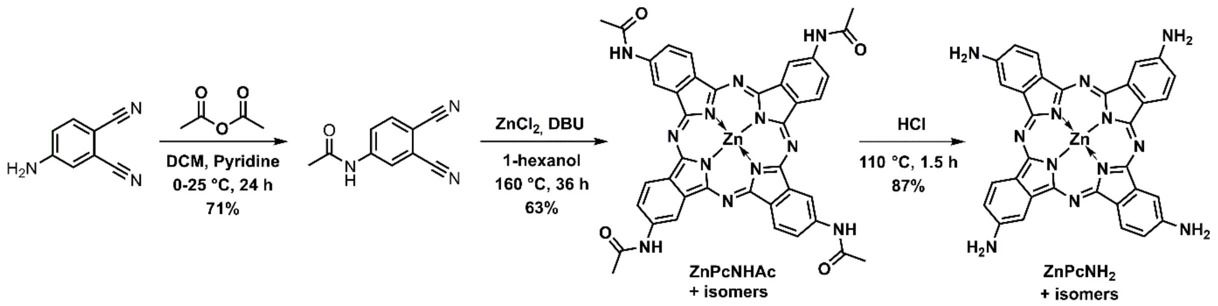

Fig.S1. Synthesis of the zinc (tetraamino)phthalocyanine (ZnPcNH$_2$).

1.1 *N*-(3,4-dicyanophenyl)acetamide

To a degassed solution of 4-aminophthalonitrile (683 mg, 143.1 g mol^{-1}, 4.77 mmol, 1 equiv) and dry pyridine (589 mg, 79.1 g mol^{-1}, 6.69 mmol, 578 μL, 1.4 equiv) in dry dichloromethane (50 mL) at 0 °C, a solution of acetic anhydride (683 mg, 102.1 g mol^{-1}, 6.69 mmol, 738 μL, 1.4 equiv) in dry DCM (18 mL) was added dropwise. The resulting mixture was stirred under argon at room temperature for 24 hours. After that, the reaction mixture was poured into a mixture of water/ice and the pH was adjusted around 4, with concentrated aqueous hydrochloric acid. The organic phase was separated and the aqueous layer was extracted with ethyl acetate. The combined organic phases were dried over anhydrous magnesium sulfate, filtered and the solvent was evaporated under reduced pressure. The

crude product was purified by column chromatography on silica gel in DCM-EA. Yield: 630 mg (185.2 g mol^{-1}, 3.40 mmol, 71%) of yellow-white powder.

IR-ATR (ν, cm^{-1}): 3305, 3274, 3180, 3119, 3101, 2232, 1709, 1679, 1581, 1526, 1493, 1405, 1369, 1321, 1261, 1236, 1202, 1095, 1009, 881, 851, 835, 722, 628, 590, 523.

^{1}H NMR (300 MHz, $CDCl_3$) δ (ppm): 10.01 (br, 1H) 8.19 (d, *J*= 2.1 Hz, 1H), 8.03 (dd, *J*= 8.7 Hz, 2.1 Hz, 1H), 7.69 (d, *J*= 8.7 Hz, 1H), 2.10 (s, 3H).

1.2 Zinc (tetraacetamide)phthalocyanine (ZnPcNHAc)

The synthesis of ZnPcNHAc was adapted from the literature:[1, 2] Under argon, anhydrous zinc chloride (458 mg, 136.3 g mol^{-1}, 3.36 mmol) was added to a degassed solution of *N*-(3,4-dicyanophenyl)acetamide (622 mg, 185.2 g mol^{-1}, 3.36 mmol) and 1,8-diazabicyclo(5.4.0)undec-7-ene (874 mg, 152.2 g mol^{-1}, 5.74 mmol, 859 μL) in anhydrous 1-hexanol (5 mL). The reaction was stirred, under argon, at 160 °C for 36 hours; at 130 °C the solution color changes from brown to green. The precipitate was filtered and washed with plenty of methanol, then washed with boiling acetone until removing the impurities were. Yield: 430 mg (806.1 g mol^{-1}, 0.533 mmol, 63%) of dark green powder.

IR-ATR (ν, cm^{-1}): 3333, 3217, 3055, 1674, 1600, 1543, 1482, 1327, 1295, 1256, 1083, 1046, 824, 743.

1.3 Zinc (tetraamino)phthalocyanine ($ZnPcNH_2$)

ZnPcNHAc (215 mg, 806.1 g mol^{-1}, 0.267 mmol) and concentrated aqueous hydrochloric acid (8 mL) were heated at 110 °C for 1.5 hours. After that, the reaction mixture was poured into a mixture of water/ice and the resulting dark precipitate was filtered off and washed with 10% aqueous sodium hydroxide, then water, ethanol, and methanol respectively. Yield: 148 mg (638.0 g mol^{-1}, 0.232 mmol, 87%) of a black-green powder.

IR-ATR (ν, cm^{-1}): 3304, 3203, 2954, 1603, 1484, 1458, 1395, 1343, 1317, 1255, 1131, 1095, 1019, 860, 814, 735.

HRMS (ESI) m/z calcd for $C_{32}H_{20}N_{12}Zn$: 636.1225 [M^+]; found: 636.1229.

2. Assignment of IR signals for $ZnPcNH_2$ monomer and $(ZnPcNH_2)_{layer}$

Tab.SI.1. Assignment of IR signals recorded for $ZnPcNH_2$ and $(ZnPcNH_2)_{layer}$

**$ZnPcNH_2$** *wavenumber (cm^{-1})*	**$(ZnPcNH_2)_{layer}$** *wavenumber (cm^{-1})*	**Assignment**
3304		N-H stretching in primary amines
	3236	N-H stretching in primary and secondary amines
3203		N-H stretching primary amines
2954	2958	sp^2 C-H stretching (ring)
1603	1605	stretching C-C in pyrrole
1484	1481	C-H bending
1458	1452	C–H in-plane bending
1420	1427	C-C and C-N asymmetric stretching in isoindole
1395	1376	Zn-C stretching, C-N-C in-plane bending
1317	1337	C-C stretching in isoindole
1255	1260	C–N stretching in isoindole
1131		C–H in-plane bending
1095	1046	C–H in-plane deformation + C-N stretching in pyrroles
1019		
860	886	Out-of-plane C-H bending
814	826	Zn-N stretching
735	735	Out-of-plane C-H bending

References

1. Yüksel, F.; Gül Gürek, A.; Lebrun, C.; Ahsen, V., Synthesis and solvent effects on the spectroscopic properties of octatosylamido phthalocyanines. *New Journal of Chemistry* **2005,** *29*, 726-732.
2. Jia, H.; Yao, Y.; Zhao, J.; Gao, Y.; Luo, Z.; Du, P., A novel two-dimensional nickel phthalocyanine-based metal–organic framework for highly efficient water oxidation catalysis. *Journal of Materials Chemistry A* **2018,** *6*, 1188-1195.
